# HLA-B*40 Allele Plays a Role in the Development of Acute Leukemia in Mexican Population: A Case-Control Study

**DOI:** 10.1155/2013/705862

**Published:** 2013-11-13

**Authors:** Javier Fernández-Torres, Denhi Flores-Jiménez, Antonio Arroyo-Pérez, Julio Granados, Alberto López-Reyes

**Affiliations:** ^1^Laboratorio de Sinovio Análisis Molecular, Instituto Nacional de Rehabilitación, Secretaría de Salud (SSA), Calzada México-Xochimilco Número 289, Tlalpan, 14389 México, DF, Mexico; ^2^Departamento de Biología, Facultad de Química, Universidad Nacional Autónoma de México (UNAM), Circuito Interior, Ciudad Universitaria, Coyoacán, 04510 México, DF, Mexico; ^3^Centro Nacional de la Transfusión Sanguínea, SSA. Avenida Othón de Mendizábal 195, Gustavo A. Madero, 07360 México, DF, Mexico; ^4^Immunogenetics Division, Instituto Nacional de Ciencias Médicas y Nutrición Salvador Zubirán (INCMNSZ), SSA. Vasco de Quiroga 15, Col. Sección XVI, Tlalpan, 14000 México, DF, Mexico

## Abstract

Among oncohematological diseases, acute lymphoid leukemia (ALL) and acute myeloid leukemia (AML) are characterized by the uncontrolled production and accumulation of blasts that can lead to death. Although the physiopathology of these diseases is multifactorial, a genetic factor seems to be at play. Several studies worldwide have shown association of ALL and AML with several alleles of the major histocompatibility complex (MHC). *Objective*. To determine gene frequencies of HLA-B alleles in Mexicans (individuals with Native American genetic background admixed with European descent) with ALL and AML. *Methods*. We compared the HLA-B alleles in 213 patients with ALL and 85 patients with AML to those present in 731 umbilical cord blood (UCB) samples as a control group; this was done by means of the PCR-SSP technique. *Results*. We found an increased frequency of the HLA-B*40 allele in ALL patients as compared to the control group (14.5% versus 9.84%, *P* = 0.003, OR = 1.67); this was particularly evident in a subgroup of young (less than 18 years old) ALL patients (*P* = 0.002, OR = 1.76); likewise, a decreased frequency of HLA-B*40 allele in AML patients was observed as compared to the control group (4.70% versus 9.84%, *P* = 0.02, OR = 0.42). *Conclusions*. These results might suggest opposing effects of the HLA-B*40 in the genetic susceptibility to develop ALL or AML and offer the possibility to study further the molecular mechanisms of cell differentiation within the bone marrow lineage.

## 1. Introduction

Acute leukemia constitutes a group of oncohematologic diseases with a multifactorial origin [1, 2]; acute lymphoid leukemia (ALL) and acute myeloid leukemia (AML) are characterized by the excessive proliferation of immature hematopoietic cells (blasts) in the bone marrow. Progressive accumulation of these cells in the peripheral blood leads to the development of fatal infections, hemorrhages, and in many cases to death [3–5].

Epidemiological studies indicate that ALL affects mainly children, whereas AML affects young adults and its incidence increases with age [5, 6]. As is the case of other Hispanic populations, ALL is the third leading cause of death in the pediatric Mexican population. Annually two to three cases per 100,000 children are reported. AML, on the other hand, is more often found in older individuals with a mean age of diagnosis of 70 years and 1 case per 100,000 adults [5, 7–10]. These high incidences can be due to the interaction of environmental, genetic, and immunologic factors, through unknown mechanisms; it has also been suggested that even recurring infections by diverse viral agents potentiate their development [11, 12].

The MHC is the most polymorphic genetic system known in humans; it is divided into class I (HLA-A, HLA-B, and HLA-C) and class II (HLA-DRB1 and HLA-DQB1) molecules, whose function is to encode membrane glycoproteins that act as mediators in the presentation of antigenic peptides to lymphocytes T CD8+ and T CD4+, respectively, thereby triggering the immune response [13].

From the genetic point of view, polymorphisms of the MHC have been the object of several studies because of their association with diverse pathologies as well as their relationship with genetic population structure and organ and tissue transplantation. These polymorphisms are due to mutations at the DNA level promoted by selective pressure, which are translated into changes in amino acid residues of the antigen-presenting molecules [13]. It has been observed that the HLA-B *locus* has the highest number of allele variants [14] because of its ability to present peptides derived from intracellular pathogens, particularly, and therefore it has become a relevant target of natural selection along human history [15]. 

The first studies on the association between hematological neoplasms and the MHC were performed in mice infected with lymphoma-inducing viruses; It was observed that the strains susceptible to leukaemogenesis possessed the H-2^k/k^ haplotype, whereas the H-2^b/b^ haplotype conferred resistance [16, 17]. These results suggest the possibility of associating human leukemia with HLA [18]. When the first studies on the association between HLA and human leukemia were made, the increase in the frequency of HLA-A2 antigen was demonstrated [19, 20]; however, the advent of new molecular techniques has allowed the identification of new allele variants of HLA class I and class II [21–24]. 

Given the relatively high incidence of acute leukemia in the Mexican population, the aim of this work was to analyze gene frequencies of HLA-B alleles in Mexican patients with ALL and AML.

## 2. Patients and Methods

### 2.1. Patients

During the 2004–2010 time period, we received 213 clinical histories of patients with ALL and 85 clinical histories with AML diagnosed, from different health care centers of Mexico, that requested HLA-compatible UCB units from the National Center of Blood Transfusion for the use in bone marrow transplantation. The patients were individuals with Native American genetic background admixed with European descent; both genders were equally represented and had no familiar kinship. To confirm diagnosis of ALL or AML, bone marrow aspirates would need to show more than 20% blast cells. As a prerequisite for the search, it was required that the request would include the HLA of the patient, preferentially typed by molecular biology.

### 2.2. Control Group

The control group was represented by 731 UCB samples which were part of the National Donor Program in Maternity Services of different states of Mexico. These units were collected with the written informed consent of the maternal donors and, in order to be included, parents were asked about family history of genetical or neoplastic disorders. All UCB samples were processed at the same blood bank and were analyzed before and after cryopreservation based on international standards; in addition, to corroborate the results obtained, repetition studies were performed prior to the transplantations. On the other hand, follow-up was carried out by means of telephone interviews with all mothers who donated their child's umbilical cord to corroborate the health status of the infants; none of the UCB units presented problems prior to the transplantation. The range of time from birth of UCB donors to the transplant was at least two years in the majority of cases.

### 2.3. Obtaining Genomic DNA from the Control Group

From the 200 *μ*L of fresh blood collected, we obtained 100 ng/*μ*L of DNA by means of a QIAamp DNA Blood Mini Kit purchased from QIAGEN, following the manufacturer's instructions.

### 2.4. DNA Amplification of the Control Group

DNA amplification to genotype the HLA-B *locus* was carried out with the A/B/DR/DQ SSP Unitray kit purchased from Invitrogen; this was done by means of the polymerase chain reaction-single specific primer (PCR-SSP) technique. PCR amplifications were performed in 25 *μ*L reaction volumes containing 100 ng genomic DNA, 50 mM Tris-HCl, 2.5 mM MgCl_2_, 20 mM each deoxynucleotide triphosphate, 10 pmol each primer, and 1 U of Taq DNA polymerase. Thermocycling parameters were as follows: denaturation at 94°C for 5 min and 30 cycles of 94°C for 30 s, 60°C for 45 s, and 72°C for 45 s, with a final extension at 72°C for 5 min. Amplified products were resolved using agarose (Invitrogen Corporation, Carlsbad, CA, USA) gel electrophoresis. Analysis for band interpretation was performed with UniMatch Plus versions 3.2 and 4.0 software.

### 2.5. Statistical Analysis

Gene frequencies of the HLA-B alleles from both study groups were calculated by direct count. For comparison between the groups, we used *χ* ^2^ analysis by using 2 × 2 contingency tables and Fisher's exact test when appropriate; *P* values less than 0.05 were considered statistically significant. For estimating risks, we employed odds ratio (OR) with a 95% confidence interval (95% CI). These calculations were performed by using the EpiInfo statistical program (version 6, Centers for Disease Control and Prevention, Atlanta, GA, USA).

## 3. Results

We studied two separate groups, one comprising 213 patients suffering ALL (59% male and 40.4% female, median age 10 years) and 85 patients affected by AML (58.8% male and 41.2% female, median age 12 years); we also studied a group of 731 UCB units as controls.  Table 1 illustrates the main demographic characteristics of patients and controls.

We identified 24 alleles of the HLA-B *locus* in the study groups.  Table 2 shows the gene frequencies of the HLA-B *locus* in patients with ALL as compared to controls. As it is readily seen, patients with ALL show increased gene frequency of the HLA-B*40 allele as compared to the control group (14.5% versus 9.84%, *P* = 0.003, OR = 1.67, CI 95% = 1.16–2.40); whereas the gene frequency of HLA-B*39 allele was lower when compared with controls (12.2% versus 17.3%, *P* = 0.004, OR = 0.61, CI 95% = 0.42–0.87).

On the other hand, Table 3 depicts the gene frequencies of the HLA-B *locus* in patients with AML. Patients showed significantly increased gene frequency of HLA-B*27 allele as compared with controls (5.29% versus 1.29%, *P* = 0.0001, OR = 4.44, CI 95% = 1.79–10.8); in contrast, in gene frequencies of HLA-B*15 and HLA-B*40, alleles were lower when compared to the control group (1.17% versus 9.23%, *P* = 0.0001, OR = 0.11, CI 95% = 0.02–0.45; 4.7% versus 9.84%, *P* = 0.02, OR = 0.42, CI 95% = 0.18–0.93, resp.).

Table 4 shows gene frequencies of HLA-B alleles in ALL and AML patients subdivided into groups of several ages; it can be seen that the role of HLA-B*40 is more relevant in patients with less than 18 years old, but it has no influence beyond that age. Additionally, the role of HLA-B*27 already described in AML is relevant regardless age.

## 4. Discussion

The major finding of this study is that the HLA-B*40 allele distinguishes genetic susceptibility of ALL from AML in the Mexican population since it is increased in the former but not in the latter. Part of this finding was already described by Cameron et al. who found association of HLA-B40 antigen in children from Trinidad with ALL [25]. Likewise, Klitz et al. found that HLA-B*40 allele is overrepresented in pediatric patients with ALL [26].

Moreover, Barion et al., who also studied genetic polymorphic variants of HLA-B *locus* in patients from Brazil, found association of HLA-B*07 allele with AML but not with ALL [27]; this would suggest that Mexicans and Brazilians have not only differences in genetic population structure but also distinct triggering factors to the development of acute leukemia. [28].

In this study, the HLA-B*39 allele was found to be significantly decreased in ALL, suggesting a protective role for this allele; nevertheless, in the study from Klitz et al., he found this allele to be associated with an increased risk to develop ALL; it would be important to study high resolution subtypes of HLA-B*39 to better define the role of this allele in the development of ALL. Finally, in patients with AML from Venezuela, Villalobos et al. found association with the HLA-A*02/-B*40/-C*03 haplotype [29].

Another relevant allele in the present study was HLA-B*15, that is significantly decreased in AML whereas HLA-B*27 allele was increased (Table 3). The study from Klitz et al., reports association of HLA-B*15 only. 

The data presented in this study might suggest that HLA allele participate in the genetic susceptibility to develop acute leukemia either directly or as genetic markers of neighboring *loci* in linkage disequilibrium with MHC genes [30, 31]. The dual role of the HLA-B*40 allele would suggest that the HLA-B molecules have a special capacity to affect the activity of some immune responses particularly those related to lymphoproliferative neoplasia triggered by intracellular pathogens [3].

Biomarkers are objectively measurable parameters, characterized as indicators of biological processes (normal or pathological) or of responses in pharmacological studies [32]. The dual effect expressed by certain biomarkers in one and the same disease can be the result of certain molecules acting at an intermediate point in the balance between inflammatory and immunomodulating processes in order to maintain homeostasis. Although, this mechanism is not clear, one way to explain it is that each allele, in particular, depicts a different affinity in binding endogenous peptides at the binding pockets of the amino acid residues, embedded in the antigen binding groove and in their further recognition [33, 34]. 

This affinity is strongly linked with the biophysical features of alleles, which vary minimally among them but are pivotal for their association with determined diseases. The change in amino acids position shown in AML such as B*27 can alter the presentation of certain antigens related to the development of some diseases, inducing a stimulus that differs from lymphocytes T [35]. Yet, an immune response can also occur if these antigens are processed within leukemic cells and are presented through HLA molecules [24]. In the present study, molecules B*27 and B*40 showed distinct patterns of genetic associations for the two types of leukemia. This could explain why there was a significant association positive of HLA-B*27 allele in AML patients but no association positive of HLA-B*40 allele and AML; this findings might also relate to the role of receptors in natural killer cells whose ligands are part of class I molecules.

In conclusion, we determined that, just like in other regions of the world, certain HLA-B alleles are associated with acute leukemia in Mexican population. Even though the results of the present study are heterogeneous, the possible mechanism may be that binding of peptides in these HLA molecules leads to an immune response, resulting in that HLA-B*40 allele seems to be a susceptibility biomarker in the development of ALL and probably a protecting biomarker in the development of AML. This might suggest that there are different genetic and immunological mechanisms that contribute to the pathogenesis of this group of diseases and offer the possibility to study further the molecular mechanisms of cell differentiation within the bone marrow lineage.

## Figures and Tables

**Table 1:** Demographic characteristics of the study groups.

Characteristic	Patients ALL	Patients AML	Controls (UCB)
	*N* = 213	*N* = 85	*N* = 731
Male	127 (59.6%)	50 (58.8%)	365 (49.9%)
Female	86 (40.4%)	35 (41.2%)	366 (50.1%)
Median age (years)	10	12	NB

District of state of origin	DF = 111 (52.1%)	DF = 46 (54.1%)	Representativite of all Mexican geographical regions.
	Nuevo León = 53 (24.8%)	Nuevo León = 17 (20.0%)	
	Puebla = 22 (10.3%)	Jalisco = 11 (12.9%)	
	Jalisco = 12 (5.6%)	Puebla = 8 (9.4%)	
	Coahuila = 5 (2.3%)	Others = 3 (3.5%)	
	Others = 10 (4.7%)		

ALL: acute lymphoid leukemia; AML: acute myeloid leukemia; UCB: umbilical cord blood; DF: federal district; NB: newborn.

**Table 2:** Gene frequencies of HLA-B alleles in patients with ALL compared with umbilical cord blood samples, as a control group.

B* allele	Patients *N* = 213		Controls *N* = 731		*P *	OR	95% CI
	*n*	g. f.	*n*	g. f.			
07	16	0.0375	60	0.0410	NS		
08	10	0.0234	40	0.0273	NS		
13	4	0.0093	14	0.0095	NS		
14	15	0.0352	55	0.0376	NS		
15	35	0.0821	135	0.0923	NS		
18	11	0.0258	42	0.0287	NS		
27	8	0.0187	19	0.0129	NS		
35	73	0.1713	296	0.2024	NS		
38	10	0.0234	15	0.0102	NS		
39	52	0.1220	254	0.1737	0.004	0.61	0.42–0.87
40	62	0.1455	144	0.0984	0.003	1.67	1.16–2.40
41	9	0.0211	13	0.0088	NS		
42	1	0.0023	9	0.0061	NS		
44	25	0.0586	75	0.0512	NS		
45	10	0.0234	18	0.0123	NS		
48	15	0.0352	57	0.0389	NS		
49	13	0.0305	29	0.0198	NS		
51	23	0.0539	86	0.0588	NS		
52	13	0.0305	40	0.0273	NS		
53	5	0.0117	17	0.0116	NS		
55	3	0.0070	2	0.0013	NS		
56	1	0.0023	2	0.0013	NS		
57	3	0.0070	15	0.0102	NS		
58	4	0.0093	6	0.0041	NS		

ALL: acute lymphoid leukemia; g. f.: gene frequency; OR: odds ratio; CI: confidence interval; NS: not significant.

**Table 3:** Gene frequencies of HLA-B alleles in patients with AML compared with umbilical cord blood samples, as a control group.

B* allele	Patients *N* = 85		Controls *N* = 731		*P*	OR	95% CI
	*n*	g. f.	*n*	g. f.			
07	11	0.0647	60	0.0410	NS		
08	5	0.0294	40	0.0273	NS		
13	3	0.0176	14	0.0095	NS		
14	7	0.0411	55	0.0376	NS		
15	2	0.0117	135	0.0923	0.0001	0.11	0.02–0.45
18	6	0.0352	42	0.0287	NS		
27	9	0.0529	19	0.0129	0.0001	4.44	1.79–10.8
35	30	0.1764	296	0.2024	NS		
37	3	0.0176	6	0.0041	NS		
38	2	0.0117	15	0.0102	NS		
39	23	0.1352	254	0.1737	NS		
40	8	0.0470	144	0.0984	0.02	0.42	0.18–0.93
41	3	0.0176	13	0.0088	NS		
44	11	0.0647	75	0.0512	NS		
45	3	0.0176	18	0.0123	NS		
48	5	0.0294	57	0.0389	NS		
49	5	0.0294	29	0.0198	NS		
50	2	0.0117	11	0.0075	NS		
51	16	0.0941	86	0.0588	NS		
52	7	0.0411	40	0.0273	NS		
53	3	0.0176	17	0.0116	NS		
55	1	0.0058	2	0.0013	NS		
57	3	0.0176	15	0.0102	NS		
58	2	0.0117	6	0.0041	NS		

AML: acute myeloid leukemia; g. f.: gene frequency; OR: odds ratio; CI: confidence interval; NS: not significant.

**Table 4:** Subgroups based on age of patients with ALL and AML, compared with umbilical cord blood samples as a control group.

Allele	ALL patients		Controls, *N* = 731		*P*	OR	95% CI
	*n*	g. f.	*n*	g. f.			
*N* = 186, ≤18 years old							
B*39	47	0.1263	254	0.1737	0.01	0.63	0.43–0.93
B*40	56	0.1505	144	0.0984	0.002	1.76	1.20–2.56
*N* = 27, >18 years old							
B*39	5	0.0925	254	0.1737	NS		
B*40	4	0.0740	144	0.0984	NS		

Allele	AML patients		Controls, *N* = 731		*P*	OR	95% CI
	*n*	g. f.	*n*	g. f.			

*N* = 52, ≤18 years old							
B*15	1	0.0096	135	0.0923	0.002	0.09	0.0–0.59
B*27	5	0.0480	19	0.0129	0.004	3.99	1.24–11.9
B*40	5	0.0480	144	0.0984	NS		
*N* = 28, >18–59 years old							
B*15	0	—	135	0.0923	NS		
B*27	3	0.0535	19	0.0129	0.01	4.5	1.0–17.5
B*40	3	0.0535	144	0.0984	NS		
*N* = 5, >59 years old							
B*15	1	0.1000	135	0.0923	NS		
B*27	1	0.1000	19	0.0129	0.01	9.37	0.18–99.9
B*40	0	—	144	0.0984	NS		

ALL: acute lymphoid leukemia; AML: acute myeloid leukemia; g. f.: gene frequency; OR: odds ratio; CI: confidence interval; NS: not significant.
